# Identification of novel putative alleles related to important agronomic traits of wheat using robust strategies in GWAS

**DOI:** 10.1038/s41598-023-36134-z

**Published:** 2023-06-19

**Authors:** Hossein Abdi, Hadi Alipour, Iraj Bernousi, Jafar Jafarzadeh, Paulo Canas Rodrigues

**Affiliations:** 1 Department of Plant Production and Genetics, Faculty of Agriculture, Urmia University, Urmia, Iran; 2 Dryland Agricultural Research Institute (DARI), Agriculture Research, Education and Extension Organization (AREEO), Maragheh, Iran; 3 Department of Statistics, Federal University of Bahia, Salvador, 40170-110 Brazil

**Keywords:** Cell biology, Computational biology and bioinformatics, Genetics, Molecular biology, Plant sciences

## Abstract

Principal component analysis (PCA) is widely used in various genetics studies. In this study, the role of classical PCA (cPCA) and robust PCA (rPCA) was evaluated explicitly in genome-wide association studies (GWAS). We evaluated 294 wheat genotypes under well-watered and rain-fed, focusing on spike traits. First, we showed that some phenotypic and genotypic observations could be outliers based on cPCA and different rPCA algorithms (Proj, Grid, Hubert, and Locantore). Hubert’s method provided a better approach to identifying outliers, which helped to understand the nature of these samples. These outliers led to the deviation of the heritability of traits from the actual value. Then, we performed GWAS with 36,000 single nucleotide polymorphisms (SNPs) based on the traditional approach and two robust strategies. In the conventional approach and using the first three components of cPCA as population structure, 184 and 139 marker-trait associations (MTAs) were identified for five traits in well-watered and rain-fed environments, respectively. In the first robust strategy and when rPCA was used as population structure in GWAS, we observed that the Hubert and Grid methods identified new MTAs, especially for yield and spike weight on chromosomes 7A and 6B. In the second strategy, we followed the classical and robust principal component-based GWAS, where the first two PCs obtained from phenotypic variables were used instead of traits. In the recent strategy, despite the similarity between the methods, some new MTAs were identified that can be considered pleiotropic. Hubert's method provided a better linear combination of traits because it had the most MTAs in common with the traditional approach. Newly identified SNPs, including rs19833 (5B) and rs48316 (2B), were annotated with important genes with vital biological processes and molecular functions. The approaches presented in this study can reduce the misleading GWAS results caused by the adverse effect of outlier observations.

## Introduction

Bread wheat (*Triticum aestivum* L.) is vital to the world's agricultural economy. Understanding the basis of its genetic and phenotypic diversity is essential to increase grain yield. Such a goal requires statistical methods with the correct application^1^. In this regard, cPCA has been one of the most important, simplest, and most widely used statistical tools for breeders. PCA aims to reduce data complexity in several principal components (PC). This multivariate method, in addition to studies of phenotypic and molecular diversity^2,3^, has been used in confirming population structure^4,5^, genotype selection^6^, understanding the pattern of genotype-by-environment interactions^7,8^, and selection of traits for yield modeling^9^. Despite its widespread use, the results of this technique can be highly biased in population genetic research^10^ and GWAS^11^, which have been cited as potential pitfalls^12^.

Most of the problems of PCA are due to the high sensitivity of this method to the presence of outliers in the data. Phenotypic and genotypic data are susceptible to outlier samples, regardless of the cause. Outlier observations have been observed in single-site and multi-environment trials^13,14^, DNA-seq^15,16^, and RNA-seq^17^ data. Therefore, the presence of such observations in the data is inevitable and violates the underlying assumptions of many statistical analyses^18^. The problem of outlier observations does not end only with their adverse effects, but identifying these observations and managing them is a very challenging task^19,20^. So far, several statistical methods have been developed to identify outliers, being the multivariate detection of outliers using PCA one of these approaches^12^. The sensitivity of cPCA to outliers can be solved by using a robust PCA (rPCA) algorithm^17,21^.

Over the past years, GWAS has proven its power in quantitative trait locus (QTL) mapping. Many researchers have tried deciphering and dissect the genetic basis of various wheat traits using this approach^22–26^. Meanwhile, various QTLs distributed in almost all chromosomes have been identified for important wheat traits such as spike weight, grain number, thousand kernel weight, and grain yield^24,25,27–31^. It was also concluded that many candidate genes for wheat grain yield under rainfed environments are in the form of gene clusters^32^. In some GWAS studies based on gene annotation, it has been determined that SNPs identified for yield and yield components under drought stress are linked to genes that play an important role in plant growth and survival^33–35^. Statistical methods are the core of GWAS^36^. Although several of these methods have been developed for GWAS, most of them are sensitive to phenotypic outliers, making their results unreliable^11^. Population structure is used as a covariate in GWAS to avoid false positive rates. Due to the high dimensionality, determining complex population substructures using SNPs is challenging^37^. PCA is used as a simple method in setting population classification in these types of studies, and usually, its first few PCs are included as covariates of the population structure in the model. This method may not be suitable for properly diagnosing the population structure in the presence of outlier data^21^. Such PCA can wrongly consider linkage disequilibrium (LD) structure instead of population structure, and principal component scores may capture outliers^12^. Therefore, the linear combinations of the first three PCs, included as covariates in the model, can affect the GWAS results. On the other hand, sometimes, instead of phenotype variables, the PC obtained from them is used for GWAS^38–41^. Therefore, the contamination of phenotypic data with outlier observations may also distort the GWAS results. In general, cleaning outliers and selecting robust statistical models in GWAS reduces the false positive and negative rates of QTL detection^42^. In addition to GWAS, it has been reported that different methods of genome-wide QTL mapping studies are also susceptible to outliers, and in their presence, they bring misleading results^43^.

This study has three specific aims: (i) to identify phenotypic and genotypic outlier observations using PCA and rPCA; (ii) to identify novel putative alleles associated with important spike traits under well-watered and rain-fed environments using GWAS; and (iii) to determine the power of PCA and rPCA linear combinations obtained from phenotypic and genotypic data to reduce the effect of outlier samples in GWAS analysis.

## Materials and methods

### Plant materials and phenotyping

A total of 294 Iranian bread wheat genotypes, including 90 cultivars released during the last century and 204 landraces collected from different locations (Supplementary Table 1), were evaluated in a randomized complete block design under well-watered (normal irrigation) and rain-fed environments. The experiments were carried out in a research farm with coordinates 50.58 E and 35.56 N and an altitude of 1112.5 m above sea level in 2019–2020. Climate information is presented in Supplementary Table 2 according to the months of the experiment. Five spikes from each genotype were randomly selected, and different traits were measured after being transferred to Urmia University. Spike weight (SW), grain number per spike (GN), grain yield (GY), thousand kernel weight (TKW), and spike internode length (SIL) were recorded. Spike internode length was obtained from the ratio of spike axis length to the number of nodes per spike^44^. The broad-sense heritability of these traits was calculated through the following equation:

$${H}_{BS}^{2}=\frac{{\delta }_{g}^{2}}{{\delta }_{p}^{2}}=\frac{{\delta }_{g}^{2}}{{\delta }_{g}^{2}+\frac{{\delta }_{ge}^{2}}{{n}_{e}}+\frac{{\delta }_{\varepsilon }^{2}}{{n}_{e}{n}_{r}}}$$

where ${\delta }_{g}^{2}$, ${\delta }_{p}^{2}$, and ${\delta }_{ge}^{2}$ are the genotypic, phenotypic, and genotype-by-environment interaction variances, respectively. ${\delta }_{\varepsilon }^{2}$ is the error variance, Also, ${n}_{e}$ and ${n}_{r}$ are the number of environments and replicates, respectively. Finally, ${H}_{BS}^{2}$ was expressed as a percentage.

### Genotyping

Iranian wheat landraces and cultivars were genotyped using the genotyping-by-sequencing (GBS) technique, the details of which have been described previously^45^. In this method, SNPs were called using the UNEAK GBS pipeline^46^, and imputation was performed in BEAGLE v3.3.273^47^ using the w7984 reference genome. For more details, refer to Alipour et al.^48^. Finally, SNPs with heterozygotes greater than 10% and minor allele frequency less than 5% were removed, and 36,000 SNPs remained for GWAS. The distribution of SNP markers in 21 chromosomes, kinship relationships, and genetic diversity indices have been reviewed in more detail in our previous reports^25,49^.

### Linkage disequilibrium (LD)

Genome-wide linkage disequilibrium (LD) analysis was checked by calculating pairwise marker allele squared correlation coefficient (*r*^2^) for all pairwise comparisons. The LD decay pattern for the whole genome and separately for A, B, and D genomes was displayed by plotting pairwise *r*^2^ values against genetic distance (bp).

### Classical and robust principal component analysis

We used classical PCA analysis and four robust PCA (Proj, Grid, Hubert, and Locantore) methods to evaluate the population structure and identify outliers. Multivariate detection of outlier observations includes examining each observation based on a combination of variables. Usually, researchers deal with more than two variables in their data, so it is necessary to determine outlier observations in terms of a combination of variables. PCA uses the distance of the sample scores as a criterion for investigating outlier observations. The greater the distance between the observed scores, the higher the possibility of outliers. Most rPCAs use the Projection-Pursuit (PP) principle. PP methods are suitable for analyzing data sets with many variables, and they aim to find structures in multivariate data by projecting them on a low-dimensional subspace. These methods maximize a robust measure of spread to obtain consecutive directions on which the data points are projected^50^. Here, the above four PCA methods are briefly described. The Grid method uses the PP technique to calculate PCA estimators, computed via a grid search algorithm in the plane rather than p-dimensional space^51^. This algorithm leads to higher amounts of explained variability. Hubert's method, called the ROBPCA algorithm, combines PP and robust covariance estimation, i.e., minimum covariance determinant (MCD) techniques, to compute the robust loadings^52^. This rPCA method is resistant to outliers in the data and uses the MCD method for low-dimensional data sets. It is also very suitable for high-dimensional data^53^. ROBPCA yields more accurate estimates of noncontaminated datasets and more robust estimates of contaminated data. In addition, the Hubert model produces a diagnostic plot that displays and classifies outliers^52^. The spherical principal components procedure, called the Locantore method, is a functional data analysis method. This method performs cPCA on the data but is projected onto a unit sphere. The Locantore approach is very fast, and the estimates of the eigenvectors are consistent. This method is intended to explore the structure of populations of complex objects^54^. Finally, the Proj approach, which is as fast as the previous method, was used. This approach includes a simple algorithm for approximating the PP-estimators for PCA whose PCs can be sequentially computed. In other words, the Proj approach uses PP to calculate the robust eigenvalues and eigenvectors without going through robust covariance estimation^53,55^. The cPCA and rPCA analyses were performed using the rrcov package in the R program^56^. We used the outlier plots provided by these methods to identify outlier observations. Also, The first and second PCs were plotted to obtain a biplot.

### Traditional single trait-GWAS

First, GWAS analysis was performed between SNP markers and average phenotypic data for each individual trait in each environment. Since the mixed linear model (MLM) is a standard method in GWAS for various wheat traits^57^ and has better control over confounding effects^25^, we used this model. In the MLM model, two covariates are used to prevent false positives: (i) matrix k, which represents kinship relations or family relatedness, and (ii) the first three PCs of cPCA, which are considered as the population structure (stratification) in the model. Simply put, the equation of this model is as follows:

$$Y=SNP+PCs+Kinship+e$$

where *Y* represents the studied trait, *SNP* provides genotypic information, *PCs* are the population structure, and the *kinship* represents the relationship between individuals in the population using genotypic information, and *e* is the residual error^57^. In the MLM model, individuals are considered random effects, and the relatedness among individuals is conveyed through a kinship matrix.

### Single trait-GWAS with robust principal component covariates

As we mentioned earlier, classical PCA may not correctly represent the population structure. Therefore, in the second GWAS scenario, we used the four described rPCA methods as population structure. This work was done with the aim of obtaining different linear combinations of genotypic data to moderate the effect of genotypic outliers on GWAS results. Because rPCAs perform better in the presence of outlier observations.

### Classical and robust principal component-based GWAS

PCs have been used instead of phenotype variables in GWAS in recent years. Since GWAS for PCs obtained from phenotypic data can be more valuable than traditional single trait-GWAS, we used two PCs instead of the traits themselves in the third scenario. The first two PCs were used because they explained more than 90% of the phenotypic changes, and the remaining PCs did not provide specific information. We also used the PCs obtained from cPCA and four rPCA methods. This work was done to reduce the effect of outlier observations from phenotypic data.

In all three scenarios above, the threshold of − log_10_ (*p*) > 3 was used to state statistically significant marker-trait associations (MTAs). Confidence intervals (CIs) for MTAs were calculated using the linkage disequilibrium (LD) decay. GWAS analyses were performed using genome association and prediction integrated tool (GAPIT) R-package^58^, but the necessary PCs were extracted through the rrcov package in the R program^56^. The quantile–quantile (Q-Q) plots of the observed and expected *P* values were plotted at − log10 (*p*) values to assess the adequacy of a fitted normal straight line to the markers. Finally, Venn diagrams were drawn using an online tool (https://bioinformatics.psb.ugent.be/webtools/Venn/) and the t-test was performed between the alleles of each SNP to compare the results.

### Gene ontology (GO)

The sequences surrounding all associated SNPs in the EnsemblPlants (http://plants.ensembl.org/index.html) database were annotated using basic local alignment search tools (BLAST) and aligned with IWGSC v2.1 (International Wheat Genome Sequencing Consortium database) reference genome^59^. After aligning the SNP sequences with the reference genome, overlapping genes with the highest percentage identity (%ID) and the lowest expected value (E-val) were selected for further processing and interpretation. Then, the ontology of genes, i.e., molecular function, biological process, and their cellular component, was extracted from the ensemble-gramene database. It should be noted that markers with almost identical sequences were filtered. Finally, the gene network was analyzed using the genes identified in the database for annotation, visualization, and integrated discovery (DAVID) bioinformatics resources (https://david.ncifcrf.gov/). This tool examines gene pathways based on the Kyoto Encyclopedia of Gene and Genomes (KEGG) enrichment analysis^60–62^ (https://www.genome.jp/kegg/; www.kegg.jp/kegg/kegg1.html).

### Complying with relevant institutional, national, and international guidelines and legislation

The authors declare that all relevant institutional, national, and international guidelines and legislation were respected.

### Permission for land study

The authors declare that all land experiments and studies were carried out according to authorized rules.

## Results

### Identification of outlier observations

Outlier samples were identified using cPCA and different rPCA methods based on phenotypic data in both environments and genotypic data (Table 1). These samples are determined based on the outlier plots provided by the rrcov R-package (outlier plots are not reported). Eight genotypes were identified as outlier samples in well-watered and rain-fed environments, and only genotype 182 was common among them. Based on genotypic data, 16 samples were outliers. Genotypes 4, 200, and 252 were outliers under well-watered using cPCA, and only genotype 252 was confirmed by other rPCA methods. However, in the rain-fed environment, all three genotypes that were identified as outliers by cPCA were also confirmed by rPCA methods. On the other hand, some outlier samples were identified only by robust methods.

**Table 1:** Multivariate detection of outlier samples based on cPCA and various rPCA methods in different datasets.

Dataset	No	Code	Type	Classic	Grid	Hubert	Locantore	Proj
Well-watered	1	4	Landrace	✓				
	2	111	Landrace			✓	✓	✓
	3	170	Landrace		✓			
	4	182	Landrace			✓		
	5	200	Landrace	✓				
	6	238	Cultivar		✓	✓		
	7	252	Cultivar	✓	✓	✓	✓	✓
	8	277	Cultivar			✓	✓	✓
Rain-fed	1	17	Landrace					✓
	2	82	Landrace	✓		✓	✓	
	3	105	Landrace	✓		✓	✓	✓
	4	182	Landrace		✓	✓		
	5	210	Cultivar	✓	✓			✓
	6	215	Cultivar			✓	✓	
	7	219	Cultivar			✓		
	8	286	Cultivar		✓			
SNP	1	5	Landrace	✓			✓	
	2	19	Landrace			✓		
	3	21	Landrace		✓			
	4	23	Landrace			✓		
	5	24	Landrace			✓		
	6	57	Landrace	✓				
	7	133	Landrace					✓
	8	145	Landrace		✓			
	9	161	Landrace		✓			
	10	166	Landrace				✓	
	11	213	Cultivar					✓
	12	219	Cultivar			✓		
	13	262	Cultivar			✓		
	14	270	Cultivar					✓
	15	276	Cultivar	✓			✓	
	16	283	Cultivar			✓		

According to the results, Hubert's approach differs from other methods, especially in genotypic data. The Hubert method divides the outlier plot into four parts (Supplementary Fig. 1). The normal samples are on the bottom left and are different from the other three outlier types because this category's score and orthogonal distances were low. The bottom right in the well-watered environment included genotypes No. 182, the rain-fed environment included genotypes No. 182 and 219, and the genotypic data included genotypes No. 19, 23, and 24, which had high score distance and low orthogonal distance. In the upper left space of the outlier plot, the genotypes in which the orthogonal distance is high but the score distance is low, are located. It is interesting to note that in the phenotypic data, we did not see an example in this area, but in the genotypic data, three genotypes (219, 262, and 283) were located there. The upper right will contain samples strongly deviated from normal samples and have high scores and orthogonal distances. We found such outlier examples only for phenotypic data. These results can help to understand the nature of outliers and determine their type.

There was a significant difference between genotypes in both environments for all traits. Also, except for SIL, the mean of traits in the rain-fed environment decreased (Supplementary Table 3). Descriptive statistics showed little changes by removing two genotypes (105 and 252) that were outliers by most methods. These changes were different from one trait to another. In SW, the mean decreased, but the genotypic variance increased. This mode for GN was increasing and decreasing, respectively. As expected, SD and δ^2^_GE_ decreased for all traits (except SIL). The amount of error variance also remained almost constant. The highest and lowest heritability were related to SIL and GY traits, respectively. Removing two outlier observations caused the heritability to increase by about 2% in some traits (Table 2).

**Table 2:** Mean, standard deviation (SD), variance components, and general heritability (H^2^_BS_) for the studied traits in two environments. δ^2^_G_, δ^2^_GE_, and δ^2^_E_ represent the genotypic, genotype-by-environment, and error variances, respectively^$^

Trait	Type^$^	Mean	SD	δ^2^_G_	δ^2^_GE_	δ^2^_E_	H^2^_BS_	*P*
Spike weight (g)	Outlier	2.1603	0.8994	0.118	0.142	0.380	51.98	***
	No Outlier	2.1576	0.8968	0.120	0.134	0.380	53.33	***
Grain number	Outlier	43.53	14.074	45.21	39.76	103.0	59.97	***
	No Outlier	43.40	13.967	44.32	38.24	102.1	60.18	***
Grain yield (g/plant)	Outlier	1.539	0.7356	0.074	0.096	0.260	50.00	***
	No Outlier	1.537	0.7335	0.076	0.092	0.260	51.35	***
Thousand kernel weight (g)	Outlier	35.022	11.483	23.9	27.4	52.0	55.84	***
	No Outlier	35.072	11.471	24.0	26.6	52.0	56.47	***
Spike internode length (cm)	Outlier	0.5478	0.0763	0.00256	0.00128	0.00197	75.34	***
	No Outlier	0.5476	0.0764	0.00257	0.00129	0.00198	75.24	***

In “no outlier”, genotypes 105 and 252, which were outliers by most methods, have been removed from the total data. *** significant at 0.001 probability levels by *F* test of genotypic variance.

### Linkage disequilibrium and population structure

**Figure 1:**
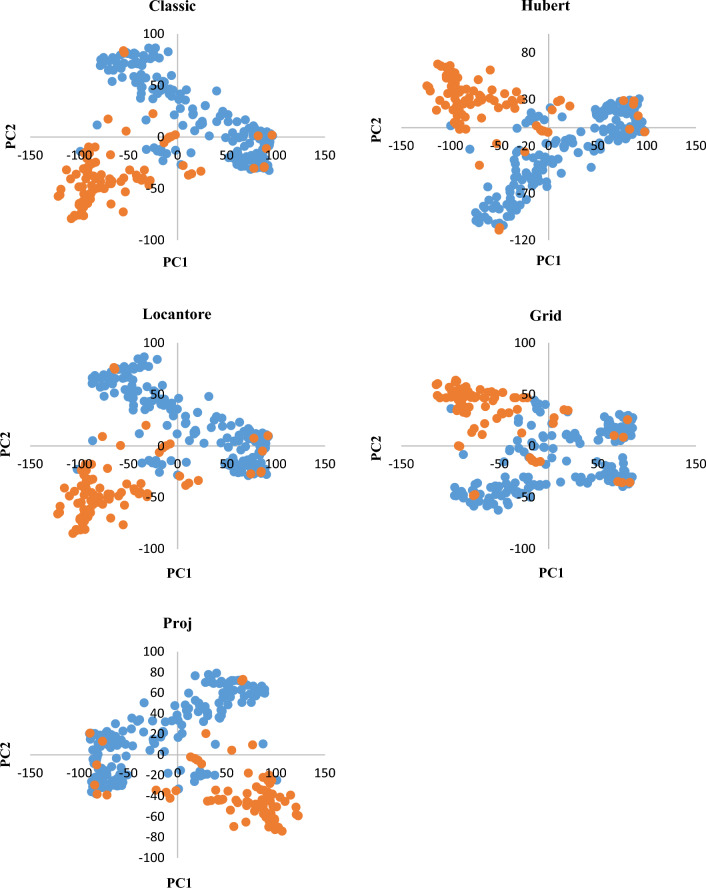
Classical and robust principal component analysis (PCA) based on 36,000 SNP markers generated from 294 Iranian wheat landraces (blue) and cultivars (orange).

The pattern of LD decay differed between sub-genomes, ranging from 1707 to 5752 bp, so its level was high in the D genome and low in the B genome (Supplementary Fig. 2). The distribution of genotypes based on the first two PCs in all methods showed a clear distinction between Iranian wheat cultivars and landraces (Fig. 1). Some cultivars were among landraces. By looking closely at the pedigree of these cultivars, we found that some of them, such as Ohadi, Roshan, and Homa, are originally landraces that have experienced various selection processes. Although, in general, the distribution pattern of genotypes was similar in all five PCA methods, it seems that the Grid approach is different from others (Fig. 1).

### Traditional single trait-GWAS

In GWAS analysis for five traits, 184 and 139 MTAs were identified in the well-watered and rain-fed environments, respectively. The details of these results are provided in Supplementary Tables 4 and 5. Briefly, In the well-watered, 38 SNPs were associated with GN, about 32% of which were located on chromosome 7A, and 90% of the markers had a significant difference between their two alleles in terms of GN (Table 3). For the same trait in rain-fed, 23 MTAs were discovered, mainly distributed in the A genome as in well-watered, and 60% of them were different between their two alleles. For SW under well-watered and rain-fed environments, respectively, we observed 19 MTAs (mostly in genome A) and 18 MTAs (mostly in genome B), of which 68% and 55% differed between their alleles in terms of average SW. Among the traits studied, most MTAs were assigned to SIL. 65 and 53 MTAs were associated with SIL in well-watered and rain-fed environments, respectively, and most of these SNPs were located on chromosomes 6B and 2B. After SIL, TKW, with 44 MTAs in a well-watered environment and 32 MTAs in a rain-fed environment, had the highest number of MTAs located in chromosome 2B. Mostly, we found a statistically significant difference between the two alleles of these markers in terms of average TKW. Finally, GY was significantly associated with 18 and 13 SNPs in well-watered and rain-fed, respectively, which were distributed in almost half of the 21 wheat chromosomes. Like the previous traits, the markers related to GY differed between their two alleles in terms of the average trait.

**Table 3:** The *t *test between two alleles of all SNPs associated with traits in two environments.

Environment	t test	Trait	Classic	Grid	Hubert	Locantore	Proj
Well-watered	Significance	SW	13 (68.4)	16 (76.2)	26 (87.7)	12 (70.6)	18 (66.7)
		GN	34 (89.5)	30 (85.7)	32 (94.1)	35 (92.1)	35 (97.2)
		TKW	42 (95.5)	39 (90.7)	35 (94.6)	46 (95.8)	39 (95.1)
		GY	11 (61.1)	17 (77.3)	26 (83.9)	12 (63.2)	11 (61.1)
		SIL	62 (95.4)	58 (96.7)	55 (96.5)	63 (95.5)	63 (92.6)
	Non-significance	SW	6 (31.6)	5 (23.8)	4 (13.3)	5 (29.4)	9 (33.3)
		GN	4 (10.5)	5 (14.3)	2 (5.88)	3 (7.89)	1 (2.78)
		TKW	2 (4.55)	4 (9.30)	2 (5.41)	2 (4.17)	2 (4.88)
		GY	7 (38.9)	5 (22.7)	5 (16.1)	7 (36.8)	7 (38.9)
		SIL	3 (4.62)	2 (3.33)	2 (3.51)	3 (4.55)	5 (7.35)
Rain-fed	Significance	SW	10 (55.6)	5 (45.5)	28 (84.8)	9 (50.0)	16 (72.7)
		GN	14 (60.9)	13 (61.9)	12 (60)	15 (62.5)	13 (68.4)
		TKW	31 (96.9)	26 (96.3)	29 (100)	28 (96.6)	34 (94.4)
		GY	10 (76.9)	6 (50.0)	17 (85.0)	10 (76.9)	13 (76.5)
		SIL	34 (64.2)	40 (69.0)	36 (69.2)	37 (63.8)	36 (70.6)
	Non-significance	SW	8 (44.4)	6 (54.5)	5 (15.2)	9 (50.0)	6 (27.3)
		GN	9 (39.1)	8 (38.1)	8 (40.0)	9 (37.5)	6 (31.6)
		TKW	1 (3.1)	1 (3.7)	0 (0)	1 (3.5)	2 (5.6)
		GY	3 (23.1)	6 (50.0)	3 (15.0)	3 (23.1)	4 (23.5)
		SIL	19 (35.8)	18 (31.0)	16 (30.8)	21 (36.2)	15 (29.4)

*SW* Spike weight, *GN* Grain number, *GY* Grain yield, *TKW* Thousand kernel weight, *SIL* Spike internode length.

### Single trait-GWAS with robust principal component covariates

**Figure 2:**
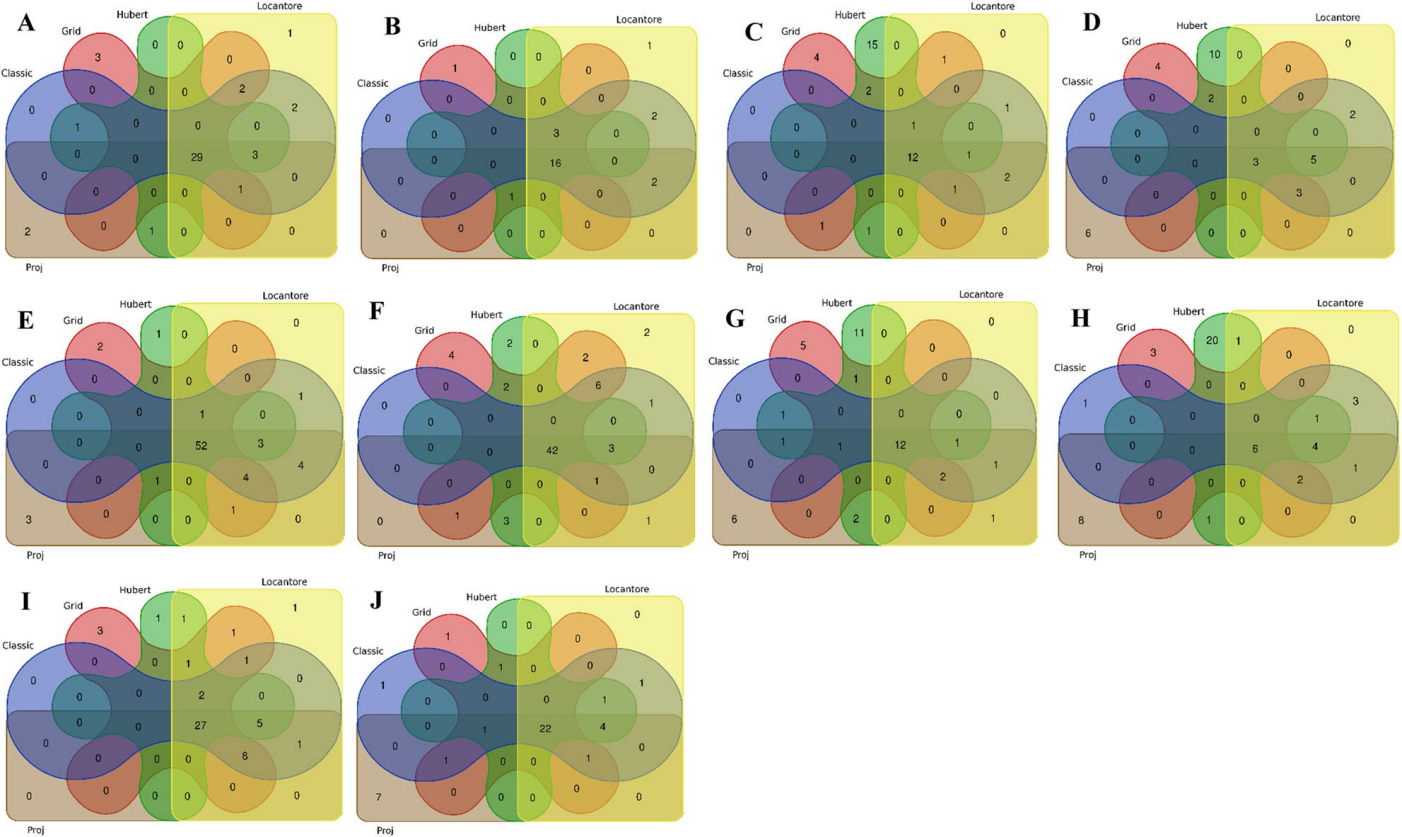
Venn plots represent the number of SNPs in classical PCA and robust PCA for evaluated traits. (**A**): grain number under well-watered, (**B**): grain number under rain-fed, (**C**): grain yield under well-watered, (**D**): grain yield under rain-fed, (**E**): spike internode length under well-watered, (**F**): spike internode length under rain-fed, (**G**): spike weight under well-watered, (**H**): spike weight under rain-fed, (**I**): thousand kernel weight under well-watered, (**J**): thousand kernel weight under rain-fed.

The details of single trait-GWAS results with different PCs as population structure are presented in Supplementary Table 4. In addition, Venn plots are drawn in Fig. 2 to show the number of common SNPs between different methods. The effect of population structure on GWAS results varied from one trait to another. In both environments, the number of common SNPs between cPCA and rPCA methods was high for GN, SIL, and TKW traits but low for traits such as GY and SW. Using the PCs provided by Hubert and Grid methods as population structure identified some new markers, especially in SW, GY, and SIL traits. Also, several new markers were identified using a covariate of the population structure by the Proj method in most traits. In some cases, the new SNPs identified by the rPCA covariate were in the same chromosomal regions as those identified by the classical method. However, others were located in new chromosomal regions that could be interesting. In Hubert and Proj's methods, new regions were found on chromosomes 7A and 6B for both SW and GY traits. Using the PCs obtained by the Locantore method, the lowest number of new MTAs was detected compared to other methods.

**Figure 3:**
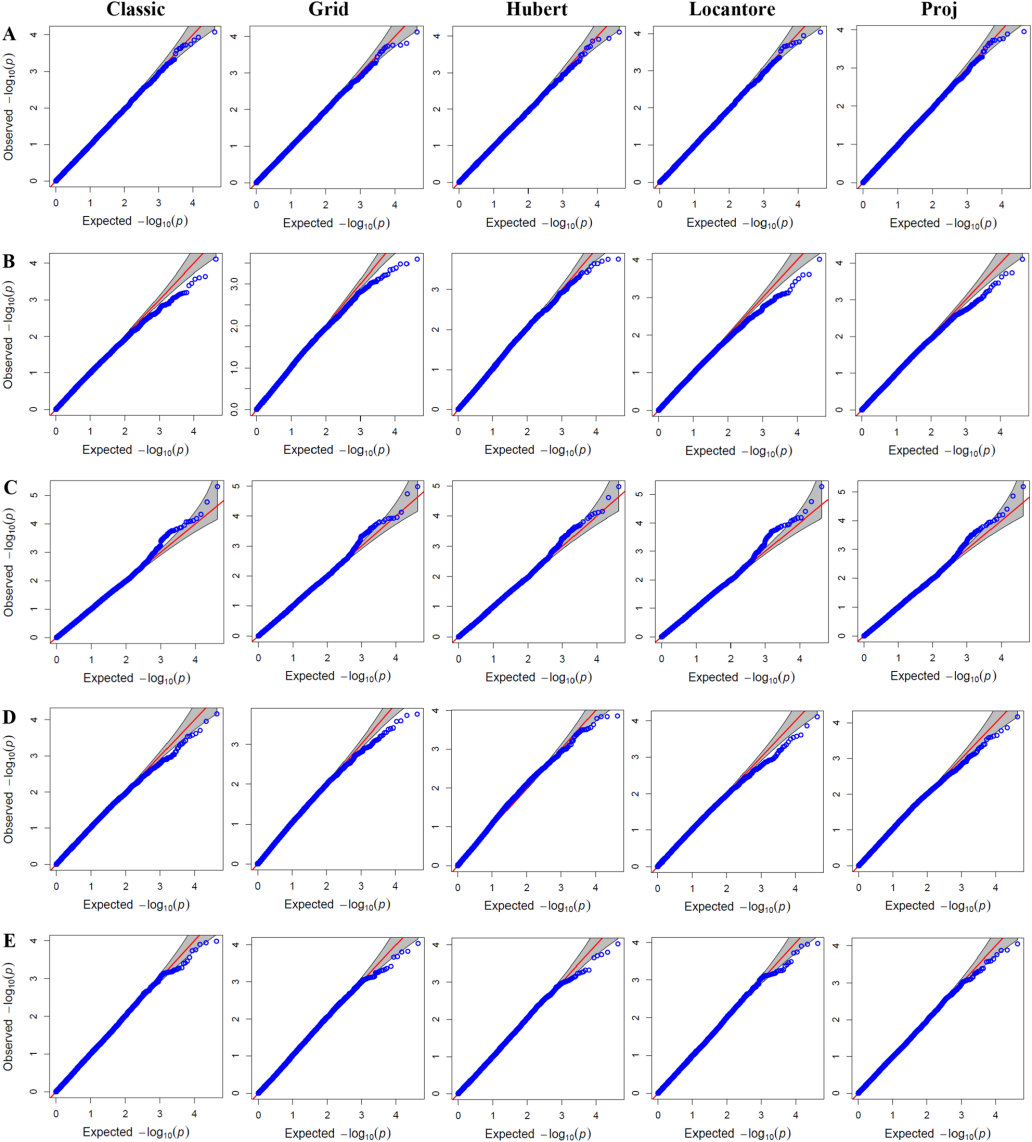
Quantile–quantile (Q–Q) plots for genome wide association study (GWAS) based on different PCA covariates in studied traits under well-watered environment. (**A**): Grain number, (**B**): Grain yield, (**C**): Spike internode length, (**D**): Spike weight, and (**E**): Thousand kernel weight.

**Figure 4:**
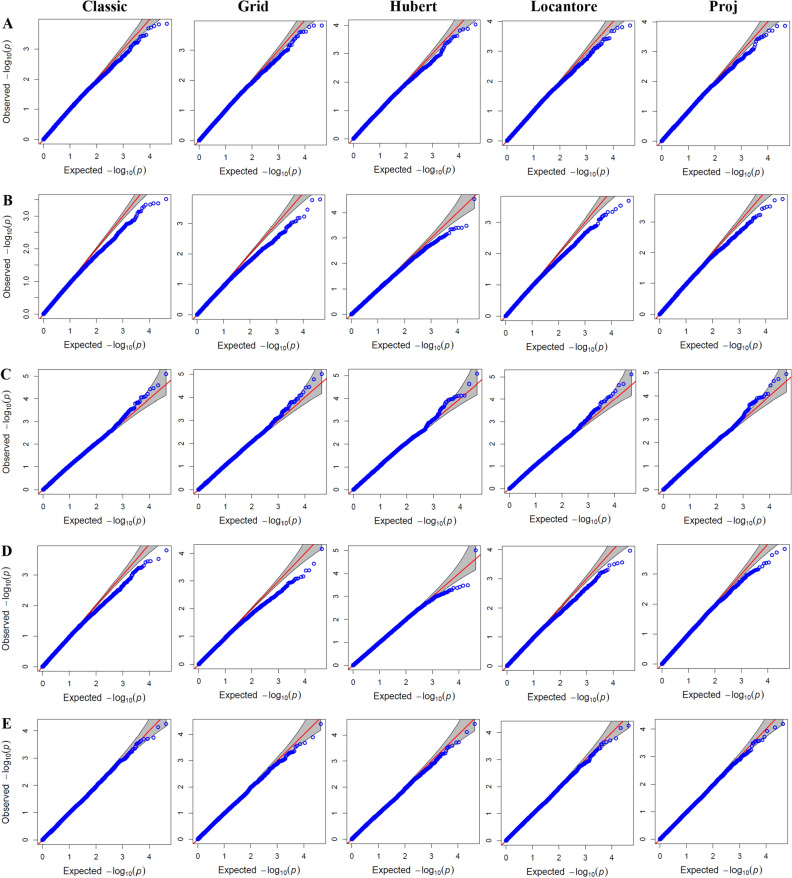
Quantile–quantile (Q–Q) plots for genome wide association study (GWAS) based on different PCA covariates in studied traits under rain-fed environment. (**A**): Grain number, (**B**): Grain yield, (**C**): Spike internode length, (**D**): Spike weight, and (**E**): Thousand kernel weight.

We evaluated the fitness and efficiency of different models with Q-Q plots. The Q-Q plots of the expected − log10 (*p*) versus the observed − log10 (*p*) for PCA covariates were almost the same in both well-watered (Fig. 3) and rain-fed (Fig. 4) environments. In general, in these methods, the observed values were close to the expected values. Only at the tail of the point distribution did we see a deviation from the expected value, indicating significant marker effects. However, minor differences are also visible; for example, the values observed in Hubert's method for GY in well-watered were closer to the expected values. Also, the p-values for GY under rain-fed seems slightly inflated, which is solved in Hubert's method.

We identified several markers associated with different traits, including two markers located in chromosomes 2A and 5A that were associated with GN, GY, and SW in a well-watered environment by all methods. Markers in chromosomes 4A, 2B, and 5B were associated with the above three traits in rain-fed. The highest number of identical markers in both environments was related to GY and SW. On the other hand, except for TKW, at least one marker was the same for the other four traits in both well-watered and rain-fed environments, which can be considered stable QTLs. As we expected, there was a significant difference between the two alleles of most of the identified SNPs in terms of the studied traits. Nevertheless, significant SNPs were high for traits such as SIL, TKW, and GN in well-watered and TKW in rain-fed. SNPs identified by Hubert and Proj methods had the highest percentage of significant markers for some traits (Table 3).

### Classical and robust principal component-based GWAS

**Figure 5:**
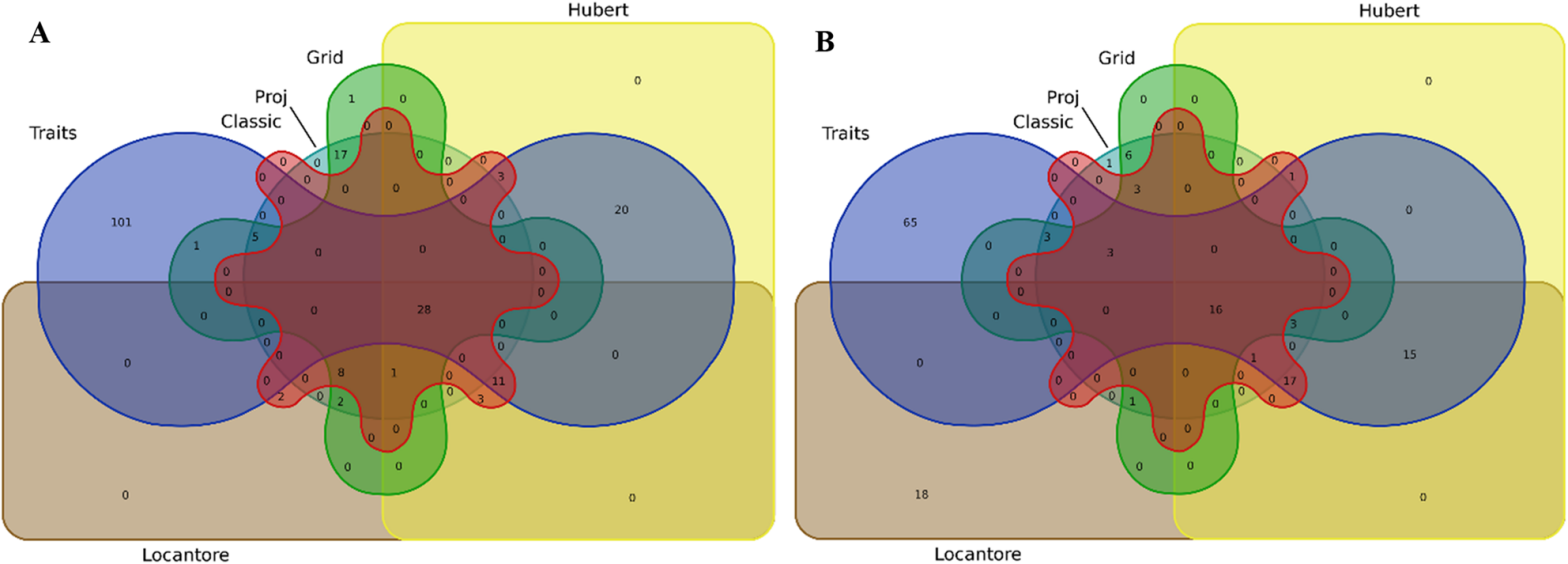
Venn plots showing the number of common markers of GWAS analysis for traits and the first two components of PCA methods in well-watered (**A**) and rain-fed (**B**) environments.

Under well-watered, 56 MTAs were discovered for the first two PCs using the classical method, while this number was 63, 66, 55, and 61 MTAs in the Grid, Hubert, Locantore, and Proj methods, respectively (Supplementary Table 5). Almost 30% of SNPs associated with PC1 in all methods were on chromosome 7A. Chromosome 2B was one of the other important chromosomes on which 20% of SNPs related to PC1 were located in Grid and Proj methods and 10% in Hubert's method. Hubert's method obtained the highest number of MTAs identified for PC2. This indicates the different linear combinations of this method compared to others. Also, Hubert's method had the highest number of common markers with traits. Almost 55% of the markers identified for traits were unrelated to any components. In contrast, 15% were observed by the components of all methods. PCs of other methods, especially Grid and Proj, discovered several MTAs that were not observed in other methods and single traits (Fig. 5A).

Such results were also obtained under drought rain-fed. Out of 139 MTAs identified for traits, 65 were absent in GWAS for the two principal components resulting from traits. The highest and lowest MTAs were assigned to PCs obtained from Locantore and Grid methods, respectively (Supplementary Table 5). In the classical method, the markers associated with PC1 and PC2 were mainly located in the A and B genomes, respectively. Unlike other methods with the most MTAs in chromosome 2B for PC2, in Grid and Proj methods, this chromosome contained more SNPs related to PC1. In Hubert's method, chromosomes 1A, 2A, 6A, and 6D had a high association with PC1, and chromosomes 2B and 6B with PC1. Almost the same results as Hubert's were obtained for two PCs from the Locantore method. Interestingly, 18 MTAs were identified for components of the Locantore method that were not present in the others. On the other hand, the SNPs associated with the Hubert method components wholly overlapped with those associated with a single trait (Fig. 5B).

### Gene annotation and Kyoto encyclopedia of genes and genomes (KEGG) pathways

Among all identified markers, 77 overlapped with a range of genes mainly located on chromosomes 2B, 3B, 6B, 1B, 7A, 4A, and 6A (Supplementary Table 6). Some of these genes had biological processes and molecular functions, forming part of cellular components such as membranes and nuclei. Defense response, protein phosphorylation, regulation of transcription, and DNA template are the most visible in biological processes. On the other hand, protein binding, ADP binding, DNA binding, iron ion binding, etc., were molecular functions of genes. The results of GO enrichment analysis and testing of statistically enriched pathways were performed in the KEGG, and some pathways, including biosynthesis of flavonoid, carotenoid, and secondary metabolites, were identified. Other pathways were metabolic, ubiquitin-mediated proteolysis, protein processing, plant-pathogen interaction, and fatty acid elongation (Table 4). Interestingly, some KEGG pathways were detected only by Hubert’s method.

**Table 4:** Kyoto encyclopedia of genes and genomes (KEGG) pathways (www.kegg.jp/kegg/kegg1.html) for overlapping genes identified by different methods.

Marker	Overlapping Gene	KEGG Pathway	Method
rs16045	TraesCS2B02G612700, TraesCS2B02G612800	Flavonoid biosynthesis, Metabolic pathways, Biosynthesis of secondary metabolites	All
rs5823	TraesCS4A02G004900	Carotenoid biosynthesis, Metabolic pathways, Biosynthesis of secondary metabolites	All
rs48469	TraesCS3B02G033700	Ubiquitin mediated proteolysis	CPC, GPC, LPC, PPC
rs48316	TraesCS2B02G098500	Inositol phosphate metabolism, Metabolic pathways, Phosphatidylinositol signaling system	CPC, GPC, PPC
rs19833	TraesCS5B02G295400	Protein processing in endoplasmic reticulum	GPC, PPC
rs62975	TraesCS4A02G191400	Phosphonate and phosphinate metabolism, Glycerophospholipid metabolism, Metabolic pathways	HPC
rs30826	TraesCS7A02G206000	Fatty acid elongation, Metabolic pathways, Biosynthesis of secondary metabolites, Plant-pathogen interaction	HPC
rs32288	TraesCS7A02G202900, TraesCS7A02G203000	Cutin, suberine and wax biosynthesis	HPC

*CPC* classical PCA, *GPC* Grid PCA, *HPC* Hubert PCA, *LPC* Locantore PCA, *PPC* Proj PCA.

## Discussion

PCA is a data dimensionality reduction method. In the analysis above, it is possible to convert many primary variables into a few new principal components, i.e., linear combinations of the original variables, that express the most variance observed in the original data. In recent years, PCA has also been used to help detect outliers. Multivariate outlier detection includes multivariate analysis of each observation based on a combination of variables^19,21,63^. Outliers samples are observations that fall outside the general distribution pattern, and contamination of data with these samples is more of a rule than an exception^53^. Outliers can be caused by various factors such as low seed quality, outlying response, misidentification of genotype, wrong data imputation, etc.^20,64^. Looking more closely at the outliers, we found that genotype No. 252, which was an outlier under well-watered by all methods, had poor seed quality and shriveled because it had high GN and low TKW. Probably, genotype No. 105 had a wrong classification in well-watered because its parameters were similar and even higher than the average of well-watered. Such deviations can have a high impact on the genotypic average^65^.

In the present study, phenotypic data compared to genotypic data, different methods identify the same samples as outliers. This is probably due to the high dimensionality in the genotypic data. Determining outliers in high-dimensional genomic data is difficult^17,20^. However, using different methods to discover them can be useful. In the outlier plot obtained from the ROBPCA algorithm (Hubert), it was found that no outlier sample had high scores and orthogonal distances based on genotypic data. If the number of accessions is high, the outlier status of some samples will change. Genotype No. 161 was present in a core set designed from among 2403 Iranian wheat landraces, and 12% of other genotypes were in outlier groups^16^. In analyzing the diversity of 80,000 wheat accessions worldwide, it was found that some are outliers. A closer examination indicated that some samples were misclassified in their passport information^66^. This is an example of the application of identifying outlier samples. Also, tools have been developed based on PCA analysis to detect outliers^67^ to, e.g., determine the SNPs under selection, and involved in biological adaptation, and have been used in various crops^15,68,69^.

A good separation of cultivars from landraces based on PCA was expected because there are traces of foreign origin in the pedigree of many Iranian wheat cultivars. Some of the cultivars mixed with the landraces are old and the detailed information about their pedigree is not available, but they are most likely of landraces origin. In this regard, Khadka et al.^4^ have discussed the possibility of mistakenly collecting exotic germplasm instead of landraces in Nepalese wheat during the 1970s to 1990s. According to our results, it has been reported that PCA based on SNPs can divide wheat accessions into separate groups according to breeding origin^32,70^. In our study, the distribution of landraces was not strictly based on their geographical origin where they were collected. The subgroups identified as population structure can represent different wheat breeding programs^33^. Therefore, using PCs as a population structure in GWAS can avoid false positive associations.

Efforts to increase wheat yields continue to meet human needs. In this regard, evaluating spike traits is critical due to their direct relationship with the grain. Several studies have emphasized the importance of spike traits^71–73^. As it was apparent, the traits studied under well-watered showed a decrease in average. SIL was an exception to this rule because the number of nodes decreased in parallel with the reduction in spike length. Heritability range varied from 50% in GY to 75% in SIL. The heritability estimated in this study for SIL is almost equal to that reported in a similar study^44^. The high heritability of this trait might be due to being mainly controlled by genetic effects. While for GY, which has complex control, heritability was low^25,74^. Our results demonstrate the distorting effect of outlier samples in estimating heritability, as previously mentioned^65^. Due to the incorrect estimation of heritability in the presence of outliers, it is suggested to use the robust estimation of heritability and to pay attention to these methods along with the classical approach^18^. In another study, the robust DF-REML framework for estimating variance components was proposed, which could provide a robust estimate of heritability^75^. However, many reports still do not pay attention to outlier data, which seems to be due to a lack of deep understanding of the distorting effects of this data.

We performed GWAS based on three approaches. Although these methods had a lot in common in highly significant MTAs, there was a difference between the methods in terms of significant SNPs at the level of − log_10_ (*p*) > 3. Such changes in GWAS results are because genomic data are rarely of good quality and are contaminated with outliers, missing values, and noise^20^. Q-Q plot is a convenient tool that shows control of type I error in MTA detection and can be used for model selection in GWAS^76–78^. Population stratification or cryptic relatedness leads to systematic deviation from the diagonal at the upper-right end of the Q-Q plots^79^. Based on this plot, there was not much difference in the results. This result is probably why we did not have outlier observation in SNP data of the third type (both score and orthogonal distances high). However, it seems that using PCs obtained from Hubert's approach can be useful in data with outlier observations of the first and second type. Also, other rPCA methods need to be investigated in diverse populations because we saw slight differences in the deviation from the diagonal at the Q-Q plots of different methods. Finally, using cPCA in genotyping data that are not contaminated by outlier observations is better.

The distribution of GY-related markers in different chromosomes has been observed before^29,80^. 4A was the chromosome that contained MTAs for GY in both environments; even SNP rs12851 on this chromosome showed its significant association in both environmental conditions. The same result was reported in similar studies that observed a stable association between chromosome 4A and yield under drought stress^81,82^. In addition, SNP rs5823, associated with GN and PC1 in all methods, was located on chromosome 4A. Interestingly, it was present in the carotenoid biosynthesis pathway and supported the cellular anatomical entity. When we used Hubert's PC for population structure, chromosome 7A was most associated with GY. According to the annotation, SNP rs30826 on this chromosome played a role in the membrane structure and was present in the plant-pathogen interaction pathway. It has been proven that there are crosstalks between responses to biotic and abiotic stresses, and some stress-resistant genes can respond to biotic and abiotic stresses^83^. Also, the plant-pathogen interaction pathway was involved in the drought stress resistance of transgenic wheat^84^. In a robust GWAS analysis, to overcome the problems of outlier observations, the linear mixed model approach was strengthened using the β-divergence method. This method performed better than the linear regression and mixed model approaches in the presence of outlier data and identified new SNPs that can be used in breeding programs^11^. The combination of GWAS and t-tests help identify significant SNPs^85^, confirming CAPS markers^86^, and identifying favorable SNP alleles^87^. Hence, we tracked the t-test for a more accurate assessment of the changes in phenotypic data due to allelic variation. The Hubert and Proj method had the highest percentage of significant SNPs in some traits compared to the classical method. The non-significant SNPs between the two alleles in terms of the t-test were distributed in most traits in a range of chromosomes; however, 2B and 7A had the highest number of these SNPs for SIL and GN traits, respectively.

Principal component-based GWAS is statistically more powerful than single-trait GWAS and requires less computational time than multi-trait GWAS. Therefore, it is an efficient method to identify pleiotropic markers^39^. If the first two PCs explain a high percentage of trait variation, they can be suitable enough for GWAS^38,40,41^. In the present study, these two PCs captured more than 90% of the variation. Chromosome 7A had a high percentage of MTAs related to PC1 in all methods. The mentioned chromosome is important due to its association with multiple agronomic traits^88^ and contains pleiotropic QTL^89,90^. Chromosome 2B was among the other regions associated with PCs in both environments. Pleiotropic loci have been reported in chromosome 2B^91^. Interestingly, under well-watered, SNP rs7805 located on chromosome 3B was associated with PC1 in all methods, while it was not significantly associated with any trait. 3B is a chromosome that simultaneously controls several yield components^25,92^. Such QTLs controlling two or more traits lead to high genetic correlation in many traits. The presence of such QTLs in the present study is not far from expected because the correlation between spike traits is certain^27,44^.

Although PC-GWAS increases statistical power and has led to the identification of new SNPs in some GWAS studies^40,41,93,94^, the presence of even a single outlier has a negative effect on PCA results^95^. The impact of outlier samples on GWAS results has already been discussed. It has been stated that the number and identity of QTLs identified through GWAS are influenced by the curation and preparation of phenotypic data. If the phenotypic data is contaminated with outliers, the number of suspicious QTLs will increase, especially in loci with unbalanced allelic frequency^64^. Therefore, it seems necessary to identify the outliers and then enter the next steps for the accuracy and precision of GWAS. For this reason, we used rPCA to obtain other linear combinations of traits that moderate the effect of outliers as input for GWAS. As far as we searched, such an approach has not been investigated in any GWAS. Under well-watered, SNP rs19833 on chromosome 5B, associated with PC1 in Grid and Proj methods, was involved in protein processing in the endoplasmic reticulum. There is evidence of the consequences of protein processing in the endoplasmic reticulum on the final grain size of wheat^96^, and we know that spike traits strongly affect the final grain size. In addition, based on the above two methods, other markers were identified that play a role in essential candidate genes, such as protein binding, protein phosphorylation, and lipid metabolic process. We saw a similar situation in the well-watered environment where SNP rs48316 located in chromosome 2B, was not associated with any trait but was significantly associated with PC1 in different methods. According to KEGG results, this SNP overlapped with the TraesCS2B02G098500 gene and was present in pathways such as the phosphatidylinositol signaling system, inositol phosphate metabolism, and metabolic pathways. All three of these pathways play a role in tolerance to drought stress^97–99^.

## Conclusion

When phenotypic and genotypic data are contaminated with outlier observations, PCA and different rPCA algorithms provided promising results in identifying them. We have shown that in such a situation, cPCA should be used cautiously in GWAS due to its sensitivity. Using robust strategies in GWAS, putative alleles for important agronomic traits in wheat were identified that were not found in conventional GWAS. Linear combinations of Hubert and Proj methods moderated the effect of phenotypic outliers to a great extent and effectively identified pleiotropic markers. Also, in GWAS results, the population structure provided by rPCA methods discovered several new QTLs associated with traits. Robust strategies in GWAS reduce the risk of missing interesting rare alleles. Finally, it is necessary to pay more attention to the effect of outlier samples and the above methods in future GWAS studies.

## Supplementary Information

Supplementary Information 1.

Supplementary Information 2.

Supplementary Information 3.

Supplementary Information 4.

## Data Availability

The datasets used and/or analysed during the current study available from the corresponding author on reasonable request.

## References

[1] Mohammadi SA, Prasanna BM (2003). Analysis of genetic diversity in crop plants—Salient statistical tools and considerations. Crop Sci..

[2] Alipour H, Abdi H (2021). Interactive effects of vernalization and photoperiod loci on phenological traits and grain yield and differentiation of Iranian wheat landraces and cultivars. J. Plant Growth Regul..

[3] Mengistu DK, Kiros AY, Pè ME (2015). Phenotypic diversity in Ethiopian durum wheat (*Triticum turgidum* var. durum) landraces. Crop J..

[4] Khadka K (2020). Population structure of Nepali spring wheat (*Triticum aestivum* L.) germplasm. BMC Plant Biol..

[5] Nielsen NH, Backes G, Stougaard J, Andersen SU, Jahoor A (2014). Genetic diversity and population structure analysis of European hexaploid bread wheat (*Triticum aestivum* L.) varieties. PLoS ONE.

[6] Godshalk EB, Timothy DH (1988). Factor and principal component analyses as alternatives to index selection. Theor. Appl. Genet..

[7] Ahakpaz F (2021). Genotype-by-environment interaction analysis for grain yield of barley genotypes under dryland conditions and the role of monthly rainfall. Agric. Water Manag..

[8] De La Vega, A. J. & Chapman, S. C. Genotype by environment interaction and indirect selection for yield in sunflower: II. Three-mode principal component analysis of oil and biomass yield across environments in Argentina. *F. Crop. Res.* **72**, 39–50 (2001).

[9] Abdipour M, Younessi-Hmazekhanlu M, Ramazani SHR, Omidi AH (2019). Artificial neural networks and multiple linear regression as potential methods for modeling seed yield of safflower (*Carthamus tinctorius* L.). Ind. Crops Prod..

[10] Elhaik E (2022). Principal Component Analyses (PCA)-based findings in population genetic studies are highly biased and must be reevaluated. Sci. Rep..

[11] Akond Z, Ahsan MA, Alam M, Mollah MNH (2021). Robustification of GWAS to explore effective SNPs addressing the challenges of hidden population stratification and polygenic effects. Sci. Rep..

[12] Prive F, Luu K, Blum MGB, Mcgrath JJ, Vilhja BJ (2020). Genetics and population analysis Efficient toolkit implementing best practices for principal component analysis of population genetic data. Bioinformatics.

[13] Tanaka, E. Simple robust genomic prediction and outlier detection for a multi-environmental field trial. arXiv Prepr. 1807.07268 (2018).

[14] Nascimento M (2021). Influential points in adaptability and stability methods based on regression models in cotton genotypes. Agronomy.

[15] Przewieslik-Allen AM (2021). The role of gene flow and chromosomal instability in shaping the bread wheat genome. Nat. Plants.

[16] Vikram P (2021). Strategic use of Iranian bread wheat landrace accessions for genetic improvement: Core set formulation and validation. Plant Breed..

[17] Chen X (2020). Robust principal component analysis for accurate outlier sample detection in RNA-Seq data. BMC Bioinf..

[18] Lourenço VM, Ogutu JO, Piepho HP (2020). Robust estimation of heritability and predictive accuracy in plant breeding: Evaluation using simulation and empirical data. BMC Genom..

[19] Pascoal C, Oliveira MR, Pacheco A, Valadas R (2010). Detection of outliers using robust principal component analysis: A simulation study. Adv. Intell. Soft Comput..

[20] Budhlakoti N, Rai A, Mishra DC (2020). Statistical approach for improving genomic prediction accuracy through efficient diagnostic measure of influential observation. Sci. Rep..

[21] Liu L, Zhang D, Liu H, Arendt C (2013). Robust methods for population stratification in genome wide association studies. BMC Bioinf..

[22] Monnot S (2021). Deciphering the genetic architecture of plant virus resistance by gwas, state of the art and potential advances. Cells.

[23] Li L (2019). Genome-wide association study reveals genomic regions controlling root and shoot traits at late growth stages in wheat. Ann. Bot..

[24] Krishnappa G (2022). Genetic dissection of grain iron and zinc, and thousand kernel weight in wheat (*Triticum aestivum* L.) using genome-wide association study. Sci. Rep..

[25] Alipour H, Abdi H, Rahimi Y, Bihamta MR (2021). Dissection of the genetic basis of genotype-by-environment interactions for grain yield and main agronomic traits in Iranian bread wheat landraces and cultivars. Sci. Rep..

[26] Li L (2019). Genetic dissection of drought and heat-responsive agronomic traits in wheat. Plant Cell Environ..

[27] Eltaher S (2022). Genome-wide association mapping revealed SNP alleles associated with spike traits in wheat. Agronomy.

[28] Wang X (2021). Genome-wide association study identifies QTL for thousand grain weight in winter wheat under normal- and late-sown stressed environments. Theor. Appl. Genet..

[29] Eltaher S (2021). GWAS revealed effect of genotype × environment interactions for grain yield of Nebraska winter wheat. BMC Genom..

[30] Khan H (2022). Genome-wide association study for grain yield and component traits in bread wheat (*Triticum aestivum* L.). Front. Genet..

[31] Zheng X (2022). Genome-wide association study of grain number in common wheat from Shanxi under different water regimes. Front. Plant Sci..

[32] Liu H (2022). Genomic regions controlling yield-related traits in spring wheat: a mini review and a case study for rainfed environments in Australia and China. Genomics.

[33] Ain QU (2015). Genome-wide association for grain yield under rainfed conditions in historical wheat cultivars from Pakistan. Front. Plant Sci..

[34] Rahimi Y, Bihamta MR, Taleei A, Alipour H, Ingvarsson PK (2019). Genome-wide association study of agronomic traits in bread wheat reveals novel putative alleles for future breeding programs. BMC Plant Biol..

[35] Rabieyan E, Bihamta MR, Moghaddam ME, Mohammadi V, Alipour H (2022). Genome-wide association mapping and genomic prediction of agronomical traits and breeding values in Iranian wheat under rain-fed and well-watered conditions. BMC Genom..

[36] Yoosefzadeh-Najafabadi, M., Eskandari, M., Belzile, F. & Torkamaneh, D. Genome-wide association study statistical models: A review. in *Genome-Wide Association Studies* (2022). 10.1007/978-1-0716-2237-7_4 35641758

[37] Chaichoompu K (2019). IPCAPS: An R package for iterative pruning to capture population structure. Source Code Biol. Med..

[38] Safdar LB (2021). Identification of genetic factors controlling phosphorus utilization efficiency in wheat by genome-wide association study with principal component analysis. Gene.

[39] Zhang W (2018). PCA-based multiple-trait GWAS analysis: A powerful model for exploring pleiotropy. Animals.

[40] Kumar K (2022). Single trait versus principal component based association analysis for flowering related traits in pigeonpea. Sci. Rep..

[41] Ma L (2021). GWAS with a PCA uncovers candidate genes for accumulations of microelements in maize seedlings. Physiol. Plant..

[42] Alvarez Prado S, Hernández F, Achilli AL, Amelong A (2022). Preparation and curation of phenotypic datasets. Methods Mol. Biol..

[43] Alam MJ, Mydam J, Hossain MR, Islam SMS, Mollah MNH (2021). Robust regression based genome-wide multi-trait QTL analysis. Mol. Genet. Genom..

[44] Wolde GM, Trautewig C, Mascher M, Schnurbusch T (2019). Genetic insights into morphometric inflorescence traits of wheat. Theor. Appl. Genet..

[45] Alipour H (2017). Genotyping-by-sequencing (GBS) revealed molecular genetic diversity of Iranian wheat landraces and cultivars. Front. Plant Sci..

[46] Bradbury PJ (2007). TASSEL: Software for association mapping of complex traits in diverse samples. Bioinformatics.

[47] Browning BL, Browning SR (2008). A unified approach to genotype imputation and haplotype-phase inference for large data sets of trios and unrelated individuals. Am. J. Hum. Genet..

[48] Alipour H (2019). Imputation accuracy of wheat genotyping-by-sequencing (GBS) data using barley and wheat genome references. PLoS ONE.

[49] Alipour H (2017). Genotyping-by-sequencing (GBS) revealed molecular genetic diversity of Iranian wheat landraces and cultivars. Front. Plant Sci..

[50] Rousseeuw, P. J. & Hubert, M. Robust statistics for outlier detection. *Wiley Interdiscip. Rev. Data Min. Knowl. Discov.* **1**, 73–79 (2011).

[51] Croux C, Filzmoser P, Oliveira MR (2007). Algorithms for projection-pursuit robust principal component analysis. Chemom. Intell. Lab. Syst..

[52] Hubert M, Rousseeuw PJ, Vanden Branden K (2005). ROBPCA: A new approach to robust principal component analysis. Technometrics.

[53] Rodrigues PC, Monteiro A, Lourenço VM (2016). A robust AMMI model for the analysis of genotype-by-environment data. Bioinformatics.

[54] Locantore N (1999). Robust principal component analysis for functional data. TEST.

[55] Croux C, Ruiz-Gazen A (2005). High breakdown estimators for principal components: The projection-pursuit approach revisited. J. Multivar. Anal..

[56] Todorov V, Filzmoser P (2009). An object-oriented framework for robust multivariate analysis. J. Stat. Softw..

[57] Saini DK (2022). Comprehensive evaluation of mapping complex traits in wheat using genome-wide association studies. Mol. Breed..

[58] Kärkkäinen HP (2012). GAPIT: Genome association and prediction integrated tool. Bioinformatics.

[59] Hosaka K (2018). Shifting the limits in wheat research and breeding using a fully annotated reference genome. Science.

[60] Ogata H (1999). KEGG: Kyoto encyclopedia of genes and genomes. Nucleic Acids Res..

[61] Kanehisa M (2019). Toward understanding the origin and evolution of cellular organisms. Protein Sci..

[62] Kanehisa M, Furumichi M, Sato Y, Kawashima M, Ishiguro-Watanabe M (2023). KEGG for taxonomy-based analysis of pathways and genomes. Nucleic Acids Res..

[63] Chiang LH, Pell RJ, Seasholtz MB (2003). Exploring process data with the use of robust outlier detection algorithms. J. Process Control.

[64] Alvarez Prado S (2019). To clean or not to clean phenotypic datasets for outlier plants in genetic analyses?. J. Exp. Bot..

[65] Ould Estaghvirou SB, Ogutu JO, Piepho HP (2014). Influence of outliers on accuracy estimation in genomic prediction in plant breeding. G3 Genes Genomes Genet..

[66] Sansaloni C (2020). Diversity analysis of 80,000 wheat accessions reveals consequences and opportunities of selection footprints. Nat. Commun..

[67] Luu K, Bazin E, Blum MGB (2017). pcadapt: An R package to perform genome scans for selection based on principal component analysis. Mol. Ecol. Resour..

[68] Skovbjerg, C. K. *et al.* Genetic analysis of global faba bean germplasm maps agronomic traits and identifies strong selection signatures for geographical origin. *bioRxiv* (2022).

[69] Bekele WA, Wight CP, Chao S, Howarth CJ, Tinker NA (2018). Haplotype-based genotyping-by-sequencing in oat genome research. Plant Biotechnol. J..

[70] Turuspekov Y (2017). GWAS for plant growth stages and yield components in spring wheat (*Triticum aestivum* L.) harvested in three regions of Kazakhstan. BMC Plant Biol..

[71] Guo Z (2018). Manipulation and prediction of spike morphology traits for the improvement of grain yield in wheat. Sci. Rep..

[72] Malik P, Kumar J, Sharma S, Sharma R, Sharma S (2021). Multi-locus genome-wide association mapping for spike-related traits in bread wheat (*Triticum aestivum* L.). BMC Genom..

[73] Zhang J (2022). Identification of genetic loci on chromosome 4B for improving the grain number per spike in pre-breeding lines of wheat. Agronomy.

[74] Godoy J (2018). Genome-wide association study of agronomic traits in a spring-planted north american elite hard red spring wheat panel. Crop Sci..

[75] Lourenço VM, Rodrigues PC, Pires AM, Piepho HP (2017). A robust DF-REML framework for variance components estimation in genetic studies. Bioinformatics.

[76] Sukumaran S, Reynolds MP, Lopes MS, Crossa J (2015). Genome-wide association study for adaptation to agronomic plant density: A component of high yield potential in spring wheat. Crop Sci..

[77] Zhou Z (2020). Identification of novel genomic regions and superior alleles associated with zn accumulation in wheat using a genome-wide association analysis method. Int. J. Mol. Sci..

[78] Aoun M, Carter AH, Ward BP, Morris CF (2021). Genome-wide association mapping of the ‘super-soft’ kernel texture in white winter wheat. Theor. Appl. Genet..

[79] Turner SD (2018). qqman: An R package for visualizing GWAS results using Q-Q and manhattan plots. J. Open Source Softw..

[80] Charity C, Mullan D, Roy S, Baumann U, Garcia M (2022). Nested association mapping-based GWAS for grain yield and related traits in wheat grown under diverse Australian environments. Theor. Appl. Genet..

[81] Shokat, S., Sehgal, D., Liu, F. & Singh, S. GWAS analysis of wheat pre-breeding germplasm for terminal drought stress using next generation sequencing technology. *Preprints* **2020020272** (2020).

[82] Ballesta P, Mora F, Del Pozo A (2020). Association mapping of drought tolerance indices in wheat: QTL-rich regions on chromosome 4A. Sci. Agric..

[83] Ku YS, Sintaha M, Cheung MY, Lam HM (2018). Plant hormone signaling crosstalks between biotic and abiotic stress responses. Int. J. Mol. Sci..

[84] Zhang Y (2021). Nucleoredoxin gene TaNRX1 positively regulates drought tolerance in transgenic wheat (*Triticum aestivum* L.). Front. Plant Sci..

[85] Xiong H (2019). A combined association mapping and t-test analysis of SNP loci and candidate genes involving in resistance to low nitrogen traits by a wheat mutant population. PLoS ONE.

[86] Wang SX (2017). Genome-wide association study for grain yield and related traits in elite wheat varieties and advanced lines using SNP markers. PLoS ONE.

[87] Su J (2019). Genome-wide association study identifies favorable SNP alleles and candidate genes for waterlogging tolerance in chrysanthemums. Hortic. Res..

[88] Jamil M (2019). Genome-wide association studies of seven agronomic traits under two sowing conditions in bread wheat. BMC Plant Biol..

[89] Chen Z (2020). Pleiotropic QTL influencing spikelet number and heading date in common wheat (*Triticum aestivum* L.). Theor. Appl. Genet..

[90] Fan X (2019). Dissection of pleiotropic QTL regions controlling wheat spike characteristics under different nitrogen treatments using traditional and conditional QTL mapping. Front. Plant Sci..

[91] Li F (2019). Genetic architecture of grain yield in bread wheat based on genome-wide association studies. BMC Plant Biol..

[92] Bonneau J (2013). Multi-environment analysis and improved mapping of a yield-related QTL on chromosome 3B of wheat. Theor. Appl. Genet..

[93] Carlson MO (2019). Multivariate genome-wide association analyses reveal the genetic basis of seed fatty acid composition in oat (*Avena sativa* L.). G3 Genes Genomes Genet..

[94] Yano K (2019). GWAS with principal component analysis identifies a gene comprehensively controlling rice architecture. Proc. Natl. Acad. Sci. USA.

[95] Alkan BB, Atakan C, Alkan N (2015). A comparison of different procedures for principal component analysis in the presence of outliers. J. Appl. Stat..

[96] Yang M (2017). Pattern of protein expression in developing wheat grains identified through proteomic analysis. Front. Plant Sci..

[97] Guo R (2018). Metabolic responses to drought stress in the tissues of drought-tolerant and drought-sensitive wheat genotype seedlings. AoB Plants.

[98] Sharma N, Chaudhary C, Khurana P (2020). Wheat Myo-inositol phosphate synthase influences plant growth and stress responses via ethylene mediated signaling. Sci. Rep..

[99] Wang X (2022). Series-temporal transcriptome profiling of cotton reveals the response mechanism of phosphatidylinositol signaling system in the early stage of drought stress. Genomics.

