# Supplementary material for: Identification of novel putative alleles related to important agronomic traits of wheat using robust strategies in GWAS
Source: Sci Rep. 2023 Jun 19;13:9927. doi: 10.1038/s41598-023-36134-z (PMC10279721; doi:10.1038/s41598-023-36134-z)
Supplement: Supplementary file 1 — Supplementary Information 1. [file 41598_2023_36134_MOESM1_ESM.docx]

**Supplementary Table 1.** Overview on the landraces and cultivars of Iranian wheat studied

| **Genetic background: Landraces** | | | | | | | | | | | | | | | |
| --- | --- | --- | --- | --- | --- | --- | --- | --- | --- | --- | --- | --- | --- | --- | --- |
| No. | USDA_PI_NO | | Region of origin (Province) | | | | |  | | No. | | USDA_PI_NO | | Region of origin (Province) | |
| 1 | | 620903 | | Azarbayjan-Gharbi | | | |  | | 47 | | 623344 | | | Bakhtaran |
| 2 | | 621420 | | Hamadan | | | |  | | 48 | | 623345 | | | Kordestan |
| 3 | | 621421 | | Hamadan | | | |  | | 49 | | 623377 | | | Kerman |
| 4 | | 621492 | | Bakhtaran | | | |  | | 50 | | 623379 | | | Kerman |
| 5 | | 621565 | | Hamadan | | | |  | | 51 | | 623382 | | | Kerman |
| 6 | | 621619 | | Kordestan | | | |  | | 52 | | 623417 | | | Sistan-Balouchestan |
| 7 | | 621650 | | Tehran | | | |  | | 53 | | 623421 | | | Azarbayjan-Shargi |
| 8 | | 621668 | | Tehran | | | |  | | 54 | | 623428 | | | Azarbayjan-Shargi |
| 9 | | 621669 | | Tehran | | | |  | | 55 | | 623473 | | | Ilam |
| 10 | | 621704 | | Gazvin | | | |  | | 56 | | 623475 | | | Ilam |
| 11 | | 621706 | | Gazvin | | | |  | | 57 | | 623503 | | | Ilam |
| 12 | | 621712 | | Gazvin | | | |  | | 58 | | 623506 | | | Bakhtaran |
| 13 | | 621716 | | Gazvin | | | |  | | 59 | | 623507 | | | Bakhtaran |
| 14 | | 621717 | | Gazvin | | | |  | | 60 | | 623508 | | | Bakhtaran |
| 15 | | 621735 | | Gazvin | | | |  | | 61 | | 623510 | | | Azarbayjan-Gharbi |
| 16 | | 621736 | | Gazvin | | | |  | | 62 | | 623905 | | | Bakhtaran |
| 17 | | 621908 | | Markazi | | | |  | | 63 | | 623908 | | | Bakhtaran |
| 18 | | 622063 | | Zanjan | | | |  | | 64 | | 623909 | | | Bakhtaran |
| 19 | | 622084 | | Mazandaran | | | |  | | 65 | | 623953 | | | Bakhtaran |
| 20 | | 622098 | | Gilan | | | |  | | 66 | | 623980 | | | Hamadan |
| 21 | | 622099 | | Gilan | | | |  | | 67 | | 624215 | | | Hamadan |
| 22 | | 622105 | | Gilan | | | |  | | 68 | | 624240 | | | Ilam |
| 23 | | 622247 | | Mazandaran | | | |  | | 69 | | 624251 | | | Ilam |
| 24 | | 622264 | | Mazandaran | | | |  | | 70 | | 624315 | | | Kordestan |
| 25 | | 622272 | | Mazandaran | | | |  | | 71 | | 624378 | | | Bakhtaran |
| 26 | | 622311 | | Khorasan | | | |  | | 72 | | 624381 | | | Bakhtaran |
| 27 | | 622379 | | Khorasan | | | |  | | 73 | | 624576 | | | Hamadan |
| 28 | | 622894 | | Esfahan | | | |  | | 74 | | 624580 | | | Hamadan |
| 29 | | 623008 | | Esfahan | | | |  | | 75 | | 624582 | | | Hamadan |
| 30 | | 623069 | | Esfahan | | | |  | | 76 | | 624585 | | | Hamadan |
| 31 | | 623090 | | Bakhtaran | | | |  | | 77 | | 624596 | | | Hamadan |
| 32 | | 623091 | | Khorasan | | | |  | | 78 | | 624804 | | | Bakhtaran |
| 33 | | 623109 | | Yazd | | | |  | | 79 | | 624805 | | | Bakhtaran |
| 34 | | 623123 | | Fars | | | |  | | 80 | | 624818 | | | Ilam |
| 35 | | 623127 | | Fars | | | |  | | 81 | | 624837 | | | Ilam |
| 36 | | 623136 | | Azarbayjan-Gharbi | | | |  | | 82 | | 624838 | | | Ilam |
| 37 | | 623139 | | Fars | | | |  | | 83 | | 624846 | | | Ilam |
| 38 | | 623161 | | Azarbayjan-Gharbi | | | |  | | 84 | | 624849 | | | Ilam |
| 39 | | 623162 | | Azarbayjan-Gharbi | | | |  | | 85 | | 624861 | | | Ilam |
| 40 | | 623169 | | Gilan | | | |  | | 86 | | 624863 | | | Ilam |
| 41 | | 623176 | | Khorasan | | | |  | | 87 | | 624864 | | | Ilam |
| 42 | | 623266 | | Azarbayjan-Gharbi | | | |  | | 88 | | 624894 | | | Kordestan |
| 43 | | 623274 | | Bakhtaran | | | |  | | 89 | | 624900 | | | Kordestan |
| 44 | | 623291 | | Hamadan | | | |  | | 90 | | 624901 | | | Kordestan |
| 45 | | 623318 | | Yazd | | | |  | | 91 | | 624910 | | | Hamadan |
| 46 | | 623338 | | Fars | | | |  | | 92 | | 624911 | | | Hamadan |
| 93 | | 624925 | | Hamadan | | | | |  | | 142 | | 626904 | | Azarbayjan-Shargi |
| 94 | | 624939 | | Tehran | | | | |  | | 143 | | 626908 | | Kerman |
| 95 | | 624941 | | Tehran | | | | |  | | 144 | | 626923 | | Gilan |
| 96 | | 624944 | | Tehran | | | | |  | | 145 | | 626924 | | Gilan |
| 97 | | 624946 | | Tehran | | | | |  | | 146 | | 626932 | | Hormozgan |
| 98 | | 624947 | | Tehran | | | | |  | | 147 | | 626933 | | Hormozgan |
| 99 | | 624956 | | Tehran | | | | |  | | 148 | | 626943 | | Kerman |
| 100 | | 624963 | | Tehran | | | | |  | | 149 | | 626958 | | Azarbayjan-Gharbi |
| 101 | | 624980 | | Gazvin | | | | |  | | 150 | | 626978 | | Esfahan |
| 102 | | 624983 | | Gazvin | | | | |  | | 151 | | 627036 | | Khouzestan |
| 103 | | 624985 | | Gazvin | | | | |  | | 152 | | 627038 | | Khouzestan |
| 104 | | 624990 | | Gazvin | | | | |  | | 153 | | 627043 | | Azarbayjan-Gharbi |
| 105 | | 625047 | | Markazi | | | | |  | | 154 | | 627054 | | Gilan |
| 106 | | 625080 | | Markazi | | | | |  | | 155 | | 627055 | | Zanjan |
| 107 | | 625081 | | Markazi | | | | |  | | 156 | | 627057 | | Gilan |
| 108 | | 625123 | | Markazi | | | | |  | | 157 | | 627061 | | Markazi |
| 109 | | 625127 | | Markazi | | | | |  | | 158 | | 627066 | | Kerman |
| 110 | | 625139 | | Markazi | | | | |  | | 159 | | 627072 | | Zanjan |
| 111 | | 625263 | | Mazandaran | | | | |  | | 160 | | 627099 | | Khouzestan |
| 112 | | 625281 | | Gilan | | | | |  | | 161 | | 627102 | | Zanjan |
| 113 | | 625362 | | Mazandaran | | | | |  | | 162 | | 627103 | | Mazandaran |
| 114 | | 625433 | | Khorasan | | | | |  | | 163 | | 627189 | | Khorasan |
| 115 | | 625661 | | Khorasan | | | | |  | | 164 | | 627236 | | Khorasan |
| 116 | | 625810 | | Khorasan | | | | |  | | 165 | | 627299 | | Yazd |
| 117 | | 626156 | | Kerman | | | | |  | | 166 | | 627356 | | Hormozgan |
| 118 | | 626158 | | Kerman | | | | |  | | 167 | | 627359 | | Markazi |
| 119 | | 626223 | | Sistan-Balouchestan | | | | |  | | 168 | | 627360 | | Kerman |
| 120 | | 626226 | | Sistan-Balouchestan | | | | |  | | 169 | | 627385 | | Bakhtaran |
| 121 | | 626234 | | Sistan-Balouchestan | | | | |  | | 170 | | 627399 | | Zanjan |
| 122 | | 626260 | | Sistan-Balouchestan | | | | |  | | 171 | | 627410 | | Azarbayjan-Shargi |
| 123 | | 626261 | | Sistan-Balouchestan | | | | |  | | 172 | | 627414 | | Bakhtaran |
| 124 | | 626358 | | Esfahan | | | | |  | | 173 | | 627416 | | Bakhtaran |
| 125 | | 626360 | | Esfahan | | | | |  | | 174 | | 627417 | | Bakhtaran |
| 126 | | 626565 | | Esfahan | | | | |  | | 175 | | 627423 | | Hamadan |
| 127 | | 626566 | | Esfahan | | | | |  | | 176 | | 627460 | | Khorasan |
| 128 | | 626573 | | Esfahan | | | | |  | | 177 | | 627484 | | Yazd |
| 129 | | 626699 | | Ilam | | | | |  | | 178 | | 627551 | | Azarbayjan-Shargi |
| 130 | | 626706 | | Hamadan | | | | |  | | 179 | | 627587 | | Kordestan |
| 131 | | 626736 | | Khorasan | | | | |  | | 180 | | 627616 | | Esfahan |
| 132 | | 626747 | | Yazd | | | | |  | | 181 | | 627688 | | Esfahan |
| 133 | | 626764 | | Yazd | | | | |  | | 182 | | 627723 | | Yazd |
| 134 | | 626776 | | Khorasan | | | | |  | | 183 | | 627760 | | Azarbayjan-Shargi |
| 135 | | 626825 | | Esfahan | | | | |  | | 184 | | 627787 | | Azarbayjan-Shargi |
| 136 | | 626846 | | Yazd | | | | |  | | 185 | | 627842 | | Kerman |
| 137 | | 626855 | | Markazi | | | | |  | | 186 | | 627845 | | Sistan-Balouchestan |
| 138 | | 626872 | | Fars | | | | |  | | 187 | | 627849 | | Sistan-Balouchestan |
| 139 | | 626881 | | Azarbayjan-Shargi | | | | |  | | 188 | | 627852 | | Sistan-Balouchestan |
| 140 | | 626883 | | Fars | | | | |  | | 189 | | 627853 | | Sistan-Balouchestan |
| 141 | | 626895 | | Azarbayjan-Shargi | | | | |  | | 190 | | 627856 | | Mazandaran |
| 191 | | 627873 | | Zanjan | | | | |  | | 198 | | 627987 | | Zanjan |
| 192 | | 627881 | | Azarbayjan-Shargi | | | | |  | | 199 | | 627990 | | Bakhtaran |
| 193 | | 627883 | | Azarbayjan-Shargi | | | | |  | | 200 | | 628012 | | Bakhtaran |
| 194 | | 627905 | | Mazandaran | | | | |  | | 201 | | 628084 | | Mazandaran |
| 195 | | 627908 | | Markazi | | | | |  | | 202 | | 628088 | | Markazi |
| 196 | | 627948 | | Markazi | | | | |  | | 203 | | 628114 | | Esfahan |
| 197 | | 627963 | | Hamadan | | | | |  | | 204 | | 628189 | | Ilam |
| **Genetic background: Cultivars** | | | | | | | | |  | |  | |  | |  |
| No. | Variety name | | | | Released year | Growth habit | Pedigree | | | | | | | | |
| 205 | 4820 | | | | 1951 | Spring | No Information | | | | | | | | |
| 206 | ADL | | | | 1976 | Spring | TK/SHAHPASSAND | | | | | | | | |
| 207 | AFLAK | | | | 2010 | Spring | HD160/5/Tob/Cno/23854/3/Nai60//Tit/Son64/4/LR/Son64 | | | | | | | | |
| 208 | AKBARI | | | | 2006 | Spring | 1-63-31/3/12300/TOB//CNO67/SX | | | | | | | | |
| 209 | AKOVA | | | | 1958 | Winter | No Information | | | | | | | | |
| 210 | ALBORZ | | | | 1978 | Spring | FN/MD//K117A/3/2*CLLF/4/SON64/KLRE/3/CNO//LR64*2/SON64 | | | | | | | | |
| 211 | ALVAND | | | | 1995 | Facultative | 1-27-6275/CF 1770  or CF17170 1-22-11 | | | | | | | | |
| 212 | ARTA | | | | 2006 | Spring | HD2206/Hork//Bul/6/CMH80A.253/2/M2A/CML//Ald*4/5/BH1146/H56.71//BH1146/3/CMH78.390/4/Seri 82/7/Hel/3*Cno79/7/2*Seri 82 | | | | | | | | |
| 213 | ARVAND | | | | 1974 | Spring | RSH/3/MTA//KY/MAYO58 | | | | | | | | |
| 214 | ATRAK | | | | 1995 | Spring | JUP/BJY'S'//URES | | | | | | | | |
| 215 | AZADI | | | | 1979 | Facultative | 4820/1.32.15409//8156 | | | | | | | | |
| 216 | AZAR1 | | | | 1957 | Winter | AZAR,LV | | | | | | | | |
| 217 | AZAR2 | | | | 1997 | Winter | KVZ/TI//MAYA/26591-1T-7M-OY-115YOM/3/SEFID | | | | | | | | |
| 218 | BAHAR | | | | 2007 | Spring | HD2172/3/BB/2*7C//Y50E/3*KAL | | | | | | | | |
| 219 | BAM | | | | 2006 | Spring | VEE#5/NAC//1-66-22   or VEERY/NACOZARI-76//1-66-22 | | | | | | | | |
| 220 | BAYAT | | | | 1976 | Spring | PUNJAB-76/CHENAB-70 | | | | | | | | |
| 221 | BEZOSTAYA | | | | 1969 | Winter | LUTESCENS17/SKOROSPELKA2 | | | | | | | | |
| 222 | BISTON | | | | 1980 | Spring | 9-36/592/PIEVE or 9-36-562/PIAVE | | | | | | | | |
| 223 | GASCOGNE | | | | 1994 | --- | No Information | | | | | | | | |
| 224 | CHAMRAN | | | | 1997 | Spring | ND/VG9144//KAL/BB/3/YACO/4/VEE#5 | | | | | | | | |
| 225 | CHAMRAN2 | | | | 2013 | Spring | Attila 50Y//Attila/Bacanora | | | | | | | | |
| 226 | DARAB1 | | | | 1980 | Spring | RSH/IRN 149(60-61)//C271 | | | | | | | | |
| 227 | DARAB2 | | | | 1995 | Spring | MAYA'S'/NAC | | | | | | | | |
| 228 | DARYA | | | | 2006 | Spring | Sha4/Chil | | | | | | | | |
| 229 | DASTJERDI | | | | 1960 | Spring | DASTJERDI | | | | | | | | |
| 230 | DAYHIM | | | | 1968 | Spring | DIADEM/ITALIAI | | | | | | | | |
| 231 | DEZ | | | | 2002 | Spring | KAUZ*2/OPATA//KAUZ | | | | | | | | |
| 232 | DN11 | | | | --- | --- | No Information | | | | | | | | |
| 233 | FALAT | | | | 1990 | Spring | KVZ/BUHO//KAL/BB | | | | | | | | |
| 234 | FONG | | | | --- | --- | No Information | | | | | | | | |
| 235 | FONTANA | | | | --- | --- | No Information | | | | | | | | |
| 236 | GAHAR | | | | 1996 | Spring | ND/VG9144//KAL/BB/3/YACO'S'/4/VEE#5 | | | | | | | | |
| 237 | QABOOS | | | | 2014 | Spring | Dryland Agricultural Research Institute | | | | | | | | |
| 238 | GHODS | | | | 1988 | Spring | RHS/5/WT/4/NOR10/K54*2//FN/3/PTR/6/OMID//KAL/BB | | | | | | | | |
| 239 | GOLESTAN | | | | 1986 | Spring | D6301/NAI60//WRM/3/CNO*2/CHR | | | | | | | | |
| 240 | HAMOON | | | | 2002 | Spring | FALAT/RSH | | | | | | | | |
| 241 | HOMA | | | | 2009 | Winter | A pure Line of Sardari | | | | | | | | |
| 242 | INIA66 | | | | 1969 | Spring | LR64/SN64 | | | | | | | | |
| 243 | KARAJ1 | | | | 1974 | Facultative | 200H/VFN/RSH | | | | | | | | |
| 244 | KARAJ2 | | | | 1974 | Winter | FA//TH/MTA/3/OMI or FA//TH/MT/3/OMID | | | | | | | | |
| 245 | KARAJ3 | | | | 1974 | Winter | DRC/MXP//ISWRN-297/3/NAI60 | | | | | | | | |
| 246 | KARIM | | | | 2011 | Spring | Triticum aestivum/Sprw “s”//CA8055/3/Baconora88 | | | | | | | | |
| 247 | KAVEH | | | | 1980 | Spring | FTA/PL | | | | | | | | |
| 248 | KAVIR | | | | 1997 | Spring | Stm/3/Kal//V543/Jit716 or SHORTIM/3/KALYANSONA//V-534/JIT-716 | | | | | | | | |
| 249 | KHAZAR1 | | | | 1974 | Spring | P4160//SN64/LR64 | | | | | | | | |
| 250 | KOOHDASHT | | | | 2002 | Spring | BB/RON//CNO67/TOTA/3/JAR | | | | | | | | |
| 251 | MAHDAVI | | | | 1995 | Spring | TI/PCH/5/MT48/3/WTE*3/NAR59/TOTA63/4/MUS | | | | | | | | |
| 252 | MAROON | | | | 1991 | Spring | AVD/PCHU/5/N10/BR21.1C//KT54B/3/NAR59/1093/4/7C | | | | | | | | |
| 253 | MARVDASHT | | | | 1999 | Spring | HD2172/BLOUDAN//AZADI | | | | | | | | |
| 254 | MIHAN | | | | 2010 | Winter | Barkat/90Zhong87 | | | | | | | | |
| 255 | MOGHAN1 | | | | 1974 | Spring | LR/N10B//3*ANE | | | | | | | | |
| 256 | MOGHAN2 | | | | 1974 | Spring | LR64A/HUAR | | | | | | | | |
| 257 | MOGHAN3 | | | | 2006 | Spring | Luan/3/V763.23/V879.c8//Pvn/4/Picus/5/Opata | | | | | | | | |
| 258 | MORVARID | | | | 2009 | Spring | MILAN/SHANGHAI-7 | | | | | | | | |
| 259 | MV17 | | | | 1993 | Winter | No Information | | | | | | | | |
| 260 | NAVID | | | | 1990 | Facultative | HYS/7C | | | | | | | | |
| 261 | NAZ | | | | 1978 | Spring | II12300//LR64A/8156/3/NOR | | | | | | | | |
| 262 | NEISHABOUR | | | | 2006 | Spring | 1-63-31/3/12300/TOB//CNO67/SX | | | | | | | | |
| 263 | NICKNEJAD | | | | 1995 | Spring | F134-71/CROW'S' | | | | | | | | |
| 264 | OFOGH | | | | 2012 | Spring | GF-gy54/Attila | | | | | | | | |
| 265 | OHADI | | | | 2010 | Winter | Selection in landraces | | | | | | | | |
| 266 | PANJAMO62 | | | | 1968 | Spring | No Information | | | | | | | | |
| 267 | PARSI | | | | 2009 | Spring | Dove"S"/Buc"S"//2*Darab1 or DOVE(SIB)/(SIB)BUCKBUCK(M-84-17)//2*DARAB | | | | | | | | |
| 268 | PISHGAM | | | | 2008 | Facultative | Bkt/90Zhong87 | | | | | | | | |
| 269 | PISHTAZ | | | | 2002 | Spring | ALVAND//ALDAN/IAS 58 | | | | | | | | |
| 270 | RASHID | | | | 1968 | Facultative | N.P.7881/AZAR 2/588 | | | | | | | | |
| 271 | RAYHANI | | | | 1942 | Spring | RAYHANI | | | | | | | | |
| 272 | RIJAW | | | | 2011 | Facultative | PATO/CAL/3/7C//BB/CNO/5/CAL//CNO/SN64/4/CNO//BAD/CHR/3/KL../6/SABALAN | | | | | | | | |
| 273 | ROSHAN | | | | 1960 | Spring | Landrace | | | | | | | | |
| 274 | SABALAN | | | | 1980 | Facultative | (908//FN/A12)1-32-4382 | | | | | | | | |
| 275 | SHAHI | | | | 1967 | Winter | SHAHI | | | | | | | | |
| 276 | SHAHPASAND | | | | 1942 | Winter | SHAHPASSAND | | | | | | | | |
| 277 | SHAHRYAR | | | | 2002 | Winter | KVZ/TI//MAYA/26591-1T-7M-OY-115Y-OM/3/1-44-21863/4/ANZA/3/PI/NAR59//HYS or KAVKAZ/TANORI-71/3/MAYA-74(SIB)//BLUEBIRD/INIA/4/KARAJ-2/5/ANZA/3/PITIC-62/NDR//HYSLOP | | | | | | | | |
| 278 | SHANGHAI7 | | | | --- | Spring | No Information | | | | | | | | |
| 279 | SHIRAZ | | | | 2002 | Spring | GV/D6301//ALD/3/AZADI or GAVILAN,MEX/D-630//(SIB)ALONDRA/3/AZADI  or ALVAND//ALDAN/IAS-58 | | | | | | | | |
| 280 | SHIROODI | | | | 1997 | Spring | ND/VG9144//KAL/BB/3/YACO/4/VEE#5 | | | | | | | | |
| 281 | SIRVAN | | | | 2012 | Spring | PRL/2*PASTOR | | | | | | | | |
| 282 | SIOSSON | | | | 1994 | --- | No Information | | | | | | | | |
| 283 | SISTAN | | | | 2006 | Spring | Bank"s"/Veery"s" | | | | | | | | |
| 284 | SIVAND | | | | 2009 | Spring | Kauz"S"/Azd or KAUZ(SIB)/(AZD)AZADI | | | | | | | | |
| 285 | SEPAHAN | | | | 2006 | Spring | AZADI/5/L2453/1347/4/KAL//BB/KAL/3/Y50E/3*KAL | | | | | | | | |
| 286 | TAJAN | | | | 1995 | Spring | BOW/NKT | | | | | | | | |
| 287 | TAKAB | | | | 2013 | Winter | Manning/Sdv1//Dogu88 | | | | | | | | |
| 288 | TOUBARI | | | | 1969 | Spring | No Information | | | | | | | | |
| 289 | TOUS | | | | 2002 | Facultative | SPN/MCD//CAM/3/NZR or  SPN/MCD//CAMA/3/NZT | | | | | | | | |
| 290 | UROUM | | | | 2009 | Winter | Alvand//Ns732/Her | | | | | | | | |
| 291 | VEE/NAC | | | | 1997 | Spring | Veery/Nacozari or Veery#5/NacozariF76 | | | | | | | | |
| 292 | ZAGROS | | | | 1996 | Spring | TAN'S'/VEE'S'//OPATA | | | | | | | | |
| 293 | ZARE | | | | 2010 | Facultative | 130L1.11//F35.70/MO73/4/YMH/TOB//MCD/3/LIRA | | | | | | | | |
| 294 | ZARRIN | | | | 1995 | Facultative | NAI60/HVII//BUC/3/F59.71/GHK | | | | | | | | |

**Supplementary Table 2.** Climatic information of the year of the experiment (2019-2020)

| **Month** | **MinT (°C)** | **MaxT (°C)** | **AT (°C)** | **TR (mm)** | **ARH (%)** | **SH** | **EV (mm)** |
| --- | --- | --- | --- | --- | --- | --- | --- |
| November | 6.383 | 17.080 | 11.52 | 0.630 | 43.479 | 6.960 | 3.189 |
| December | 1.652 | 12.303 | 6.671 | 4.710 | 50.419 | 7.226 | 0 |
| January | -0.055 | 9.0770 | 4.052 | 19.84 | 54.476 | 6.526 | 0 |
| February | 2.039 | 10.739 | 6.464 | 31.73 | 64.755 | 5.829 | 0 |
| March | 8.377 | 20.558 | 14.652 | 14.11 | 38.952 | 7.303 | 0 |
| April | 7.793 | 19.983 | 13.633 | 45.81 | 51.413 | 7.563 | 6.714 |
| May | 12.061 | 25.513 | 18.432 | 57.07 | 54.907 | 8.287 | 6.161 |
| June | 17.347 | 33.807 | 25.583 | 7.230 | 37.492 | 11.10 | 11.143 |
| *MinT* Minimum temperature, *MaxT* Maximum temperature, *AT* Average temperature, *TR* Total rainfall, *ARH* Average relative humidity, *SH* Sunny hours, *EV* Evaporation | | | | | | | |

**Supplementary Table 3.** Descriptive statistics and *F* test for studied traits in well-watered and rain-fed environments

| **Abb.** | **Trait** | **Well-watered** | | | |  | **Rain-fed** | | | |
| --- | --- | --- | --- | --- | --- | --- | --- | --- | --- | --- |
|  |  | **Min** | **Max** | **Mean** | ***P*** |  | **Min** | **Max** | **Mean** | ***P*** |
| SW | Spike weight (g) | 1.01 | 4.80 | 2.57 | *** |  | 0.70 | 3.74 | 1.75 | *** |
| GN | Grain number | 22.4 | 81.8 | 46.7 | *** |  | 17.4 | 75.8 | 40.4 | *** |
| GY | Grain yield (g/plant) | 0.71 | 3.65 | 1.87 | *** |  | 0.35 | 2.84 | 1.21 | *** |
| TKW | Thousand kernel weight (g) | 20.9 | 57.3 | 40.4 | *** |  | 13.1 | 57.0 | 29.7 | *** |
| SIL | Spike internode length (cm) | 0.30 | 0.75 | 0.54 | *** |  | 0.36 | 0.77 | 0.55 | *** |
| *** significant at 0.001 probability levels by *F* test of genotypic variance. | | | | | | | | | | |

**A**

**B**

**C**

**Supplementary Figure 1.** Outlier plots in Hubert's principal component analysis method under well-watered (A), rain-fed (B), and genotypic data (C)


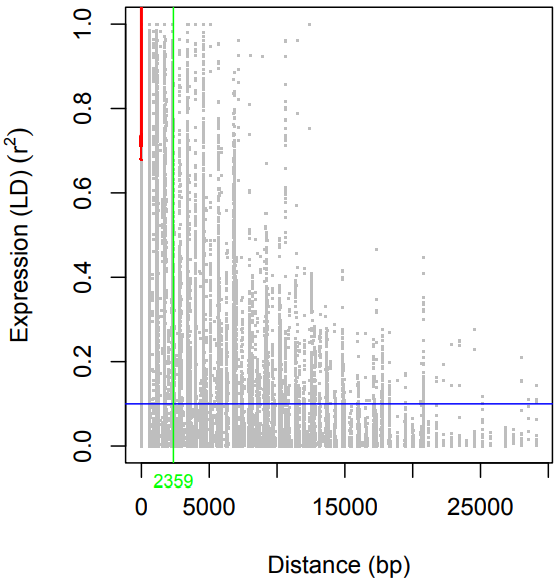

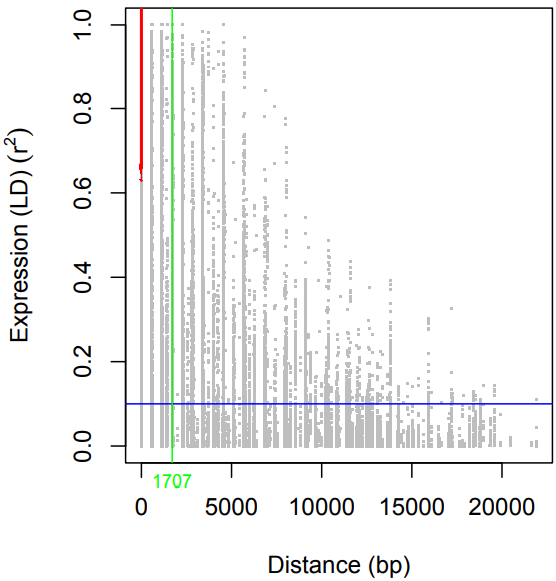


**B Genome**

**A Genome**


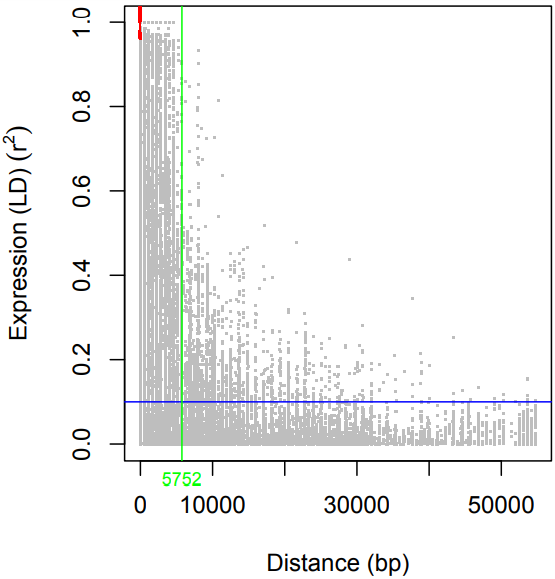

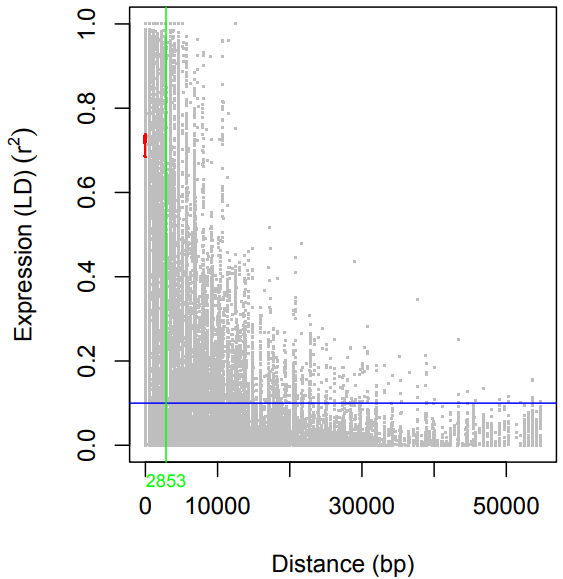


**Whole-Genome**

**D Genome**

**Supplementary Figure 2.** LD-decay plot against distance base pair by different genomes and whole-genome
